# COVID-19 and ECMO: the interplay between coagulation and inflammation—a narrative review

**DOI:** 10.1186/s13054-020-02925-3

**Published:** 2020-05-08

**Authors:** Mariusz Kowalewski, Dario Fina, Artur Słomka, Giuseppe Maria Raffa, Gennaro Martucci, Valeria Lo Coco, Maria Elena De Piero, Marco Ranucci, Piotr Suwalski, Roberto Lorusso

**Affiliations:** 1 Clinical Department of Cardiac Surgery, Central Clinical Hospital of the Ministry of Interior and Administration, Centre of Postgraduate Medical Education, Wołoska 137 Str, 02-507 Warsaw, Poland; 2 Cardio-Thoracic Surgery Department Heart and Vascular Centre, Maastricht University Medical Centre, Maastricht, the Netherlands; 3 Thoracic Research Centre Collegium Medicum, Nicolaus Copernicus University, Innovative Medical Forum, Bydgoszcz, Poland; 4 Department of Cardiovascular Anesthesia and ICU, IRCCS Policlinico San Donato, Milan, Italy; 5 Chair and Department of Pathophysiology Nicolaus Copernicus University, Collegium Medicum, Bydgoszcz, Poland; 6 Cardiac Surgery Unit, IRCCS-ISMETT, Palermo, Italy; 7 Anesthesia and Intensive Care Department, IRCCS-ISMETT, Palermo, Italy; 8 Department of Anesthesia-Intensive Care San Giovani Bosco Hospital, Turin, Italy; 9 Cardiovascular Research Institute Maastricht (CARIM)l, Maastricht, the Netherlands

## Abstract

Infection with severe acute respiratory syndrome coronavirus 2 (SARS-CoV-2) has presently become a rapidly spreading and devastating global pandemic. Veno-venous extracorporeal membrane oxygenation (V-V ECMO) may serve as life-saving rescue therapy for refractory respiratory failure in the setting of acute respiratory compromise such as that induced by SARS-CoV-2. While still little is known on the true efficacy of ECMO in this setting, the natural resemblance of seasonal influenza’s characteristics with respect to acute onset, initial symptoms, and some complications prompt to ECMO implantation in most severe, pulmonary decompensated patients. The present review summarizes the evidence on ECMO management of severe ARDS in light of recent COVID-19 pandemic, at the same time focusing on differences and similarities between SARS-CoV-2 and ECMO in terms of hematological and inflammatory interplay when these two settings merge.

## SARS-CoV-2 and COVID-19

COVID-19 is a disease caused by the novel SARS-CoV-2 virus which appeared in December 2019 and is now a worldwide pandemic [1]. Although most COVID-19 patients have moderate symptoms and recover quickly, some patients develop severe respiratory failure requiring intensive care unit (ICU) admission and, often, mechanical ventilation [2].

SARS-CoV-2 enters target cells via the angiotensin-converting enzyme 2 (ACE2) by a receptor-mediated endocytosis [3]. ACE2 is a type I integral membrane protein with several physiologic functions, well expressed in the lungs (overexpressed in smokers), heart, kidney, and gastrointestinal tract. Through the renin–angiotensin system (RAS), the virus may impact the lung circulation, but the expression on the endothelium may lead to its activation and further systemic damage with a prothrombotic state. The variable involvement of the endothelium, as well as other key organs (the liver as first), may explain the heterogeneity of the clinical picture. But as much as now is evident by the shared reports worldwide, the prothrombotic state is common in non-survivors of COVID-19 [4].

Beyond ventilator support, as well as support of other organ failures (the liver, kidney, and heart frequently involved), several drugs (antivirals, antimalaric, antibiotics, and drugs active on specific inflammatory pathways) are currently being tested but consensus or recommendations for any antiviral drug or drug combination is still lacking [3]. Several therapeutic strategies are proven to be partially ineffective, even burdened by relevant too many side effects. In the current limited health resources scenario, it would be important to adopt any adjuvant therapies that may contribute to a better outcome, but considering the impact on inflammation of the large extracorporeal surface in contact with the blood, several specific considerations should be made on the coagulation profile of the single patient [5].

## COVID-related severe respiratory impairment and V-V ECMO

The mortality in COVID-19 patients who develop severe respiratory compromise and require mechanical ventilation is high [3]. The above is of particular importance given the potential accessibility to veno-venous extracorporeal membrane oxygenation (V-V ECMO) since it may serve as life-saving rescue therapy. While still little is known on the true efficacy of ECMO in the COVID-19 setting, the natural resemblance of seasonal influenza’s complications with respect to acute onset and symptoms prompt to ECMO implantation in most severe, pulmonary decompensated patients.

**Fig. 1:**
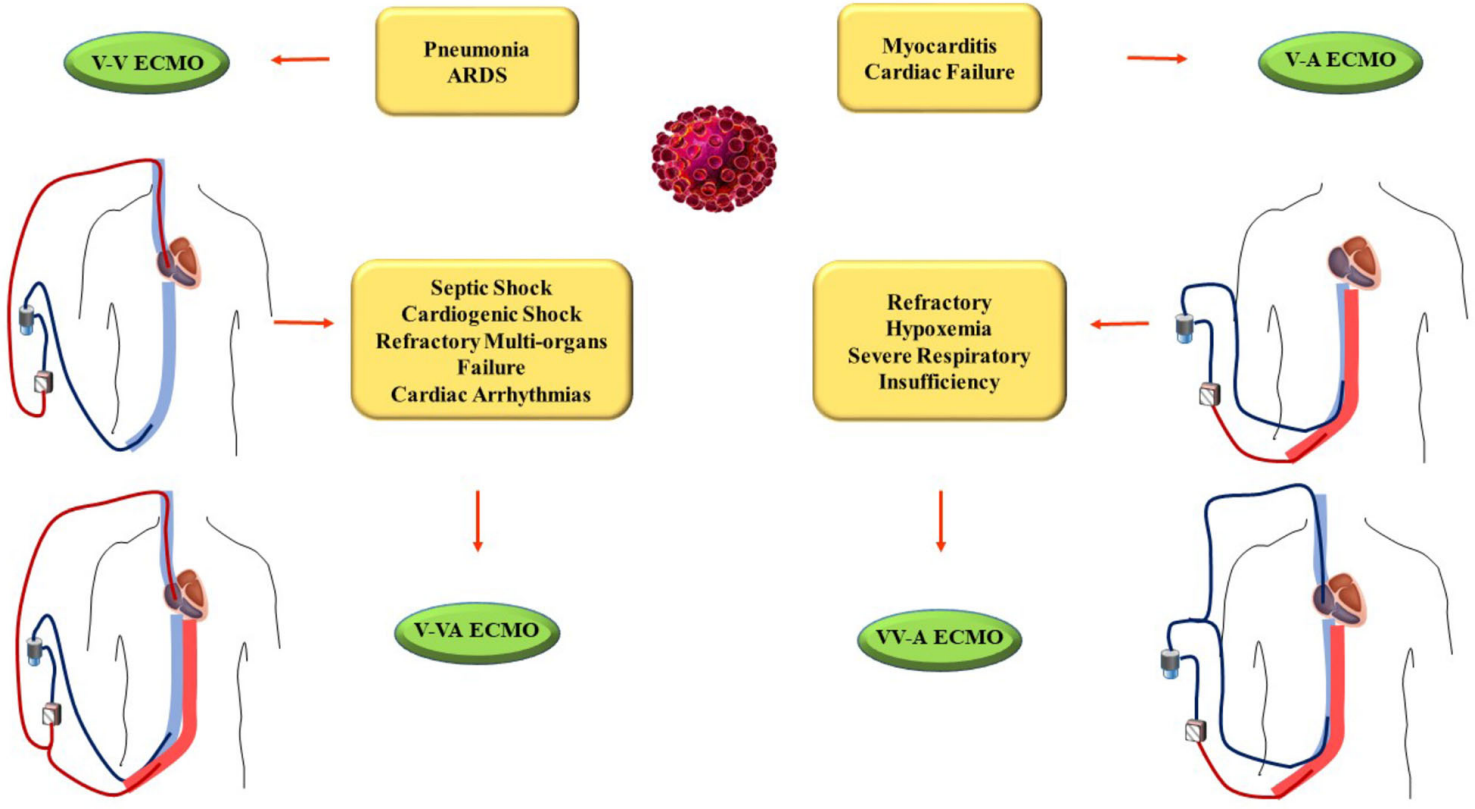
Possible ECMO configurations in COVID-19; ARDS, acute respiratory distress syndrome; ECMO, extracorporeal membrane oxygenation; V-A, veno-arterial; V-V, veno-venous; V-VA, veno-venoarterial; VV-A, venovenous-arterial

The first scenario (Fig. 1) in which ECMO may be indicated in COVID-19 patients is a severe pneumonia with acute respiratory compromise refractory to optimal conventional management including standard lung-protective ventilation strategy, prone positioning, neuromuscular blockade, and volume optimalization [6–8]. In this particular case, a V-V ECMO is indicated and the criteria to follow for its implantation are PaO2/FiO2 < 100 mmHg and/or arterial blood PH < 7.2 and PaCO2 > 60 mmHg [9]. Additional parameters to take into account may be mechanical ventilation < 7 days, age < 65 years old, ventilator frequency < 35 breath per minute (bpm), and plateau pressure > 30 cm H_2_O [10]. Some studies showed that an early use of V-V ECMO in respiratory distress may minimize respiratory-driven pressure and reduce pulmonary and systemic inflammation as well as severe multi-organ dysfunction [11, 12]. Therefore, V-V ECMO is a feasible option in COVID patients not responding to conventional interventions resulting in improved outcome and lung protection [13].

Another possible scenario is a severe myocarditis (Fig. 1) which may evolve in a cardiac dysfunction. In addition, the virus can also exacerbate comorbidities leading to ischemic heart failure as well as precipitate sepsis-related cardiomyopathy or frank septic shock-related situation. All of these conditions may need a veno-arterial (V-A ECMO) implanted as primary support [14, 15].

ECMO management is complex and dynamic according to the onset of complications and pathological events. In that way, a V-V ECMO in COVID-19 patients may also be complicated by septic and cardiogenic shock with refractory multi-organ failure as well as cardiac arrhythmias which may need an adjustment in the primary configuration [16]. In such condition, optimal biventricular unloading with concomitant high-flow ECMO support as well as partial lung perfusion with oxygenated blood may be required and achieved by adding an extra outflow cannula resulting in a veno-venoarterial (V-VA) ECMO. A hybrid ECMO may also be required when the V-A ECMO is the first approach for the development of a severe respiratory insufficiency with refractory hypoxemia. This condition may be solved with the implementation of an extra inflow cannula to improve the oxygenation (higher drainage and thus higher perfusion flow) and the metabolic needs resulting in a venovenous-arterial (VV-A) ECMO. Other situations that may require configuration alterations are the development of site- or access-related complications (i.e., bleeding of the vascular site or differential oxygenation respectively).

In COVID-19 patients, ECMO may represent an efficient support in case of severe and refractory respiratory dysfunction and/or cardiogenic/septic shock unresponsive to maximal therapy. Yet available reports focusing on respiratory compromise treatment in COVID-19 patients, and in particular, those undergoing V-V ECMO therapy however are very restrained in declaring ECMO benefit [3]. It has become evident from the published evidence that SARS-CoV-2 infection itself promotes immunological response unseen with seasonal influenza [17]; similarly, changes observed in hematological and coagulation characteristics of affected patients differ from those observed in seasonal flu infections; on top of ECMO therapy, this may, in turn, lead to unpredictable physiopathological changes in both immune and hemostatic systems, complicating the course of disease.

## Cytokine storm

Cytokine storm syndrome [18] is a hyperinflammatory state that is characterized by fulminant multi-organ failure and elevation of cytokine levels. The underlying pathophysiology of inflammatory disease may result in pulmonary, cardio-circulatory, or combined disturbances with vasodilatation and membrane leakage, which can ultimately lead to severe vasoplegic shock difficult to control. At the same time, during ECMO, the large and continuous contact surface between the humoral and cellular components of the blood and the extracorporeal circuit causes a systemic activation of coagulation and inflammation pathways that, in extreme conditions, may lead to thrombosis and disseminated intravascular coagulation [5]. The two principal mechanisms of activation of coagulation are supraphysiological shear stress and interactions between the foreign material and blood components [19–22]. The latter creates an inflammatory reaction, as already reported in the systemic inflammatory response syndrome, that involves leukocytes, platelet endothelial cells, intrinsic and extrinsic coagulation, cytokines, and complement system [23]. The result is an unbalance between pro-coagulant and anticoagulant factors, e.g., fibrinogen contributes to thrombus formation, while loss of high molecular vWF causes a bleeding tendency [24].

Infection triggers a complex host response, in which pro-inflammatory and anti-inflammatory mechanisms may contribute to the clearance of infection and tissue recovery, as well as to organ injury and secondary infections [25]. The immune reaction depends on the patient’s individual condition, coexisting diseases, and the specific load and pathogenicity of the causative agent [26, 27]. A recent study showed that COVID-19 is associated with a cytokine elevation profile that is reminiscent of secondary hemophagocytic lymphohistiocytosis [28]. All these observations may justify a wide activation of coagulation through inflammatory pathways, with a direct clinical warning about thrombosis and circuit consumption which availability and sparing may become critical during a pandemic burden.

Finally, as much of the coagulation components play a double role also in innate immunity and inflammation, also thrombin (activated factor II) is a crucial pro-coagulant protease able to initiate thromboembolic and pro-inflammatory responses [20]. In the continuous endothelial activation, antithrombin (AT) plays a relevant role, since it is more exposed on the endothelium when the cells are activated, and it is more released in the blood with consequent relatively rapid consumption in case of use of high doses of heparin [29].

The pathogenesis of COVID-19 is still under the hypothesis, but if the endothelial activation is crucial, as supposed by the presence of ACE2 on the endothelial surface, AT, as well as heparin and generally anticoagulation, may play a relevant role in reducing the inflammation and potentially mortality. Indeed, the interaction of AT with heparin-like GAGs on the endothelial cell surface involves the release of prostacyclin, which inhibits leukocyte activation by decreased release of IL-6, IL-8, and tumor necrosis factor (TNF) [30]. All these appear to be elevated during COVID-19 and it is cytokine storm that is seen at the beginning of the disease. Ruan et al. [31] and Zhou et al. [32] have identified a high level of IL-6 as a potential predictor of fatal outcome when compared between survivors and patients who died of COVID-19 disease. IL-6 is well known being linked to the trans-signaling pathway, which causes vascular leakage [33], the first step of a cascade followed by tissue edema, hypoxia, and finally necrosis. In this scenario, hemoadsorption therapy may be used to decrease cytokine levels in case of excessive inflammatory response and due to its unspecific adsorptive characteristics also substances like myoglobin, free hemoglobin, or bilirubin. Trager et al. [34] report successful treatment of septic shock and a severe SIRS response with pronounced hypercytokinemia with a combination of cytokine reduction and inflammation reduction with CytoSorb, VA-ECMO, renal support with continuous renal replacement therapy (CRRT) and low-dose hydrocortisone [35]. It seems that controlling pro-inflammatory response may be advantageous for the maintenance of the vascular barrier function, which plays a pivotal role in the development of tissue edema and oxygen mismatch.

Hemoadsorption and also other blood purification techniques can be used as stand-alone or in combination with extracorporeal circuits [25]. CRRT and ECMO yet still no sound recommendation for clinical use are made in the management of sepsis and septic shock because high-class evidence is lacking [27]. COVID-19 mortality [3] might be due to virus-activated cytokine storm syndrome, and for this reason, a novel device for adsorbing inflammatory and other mediators from the circulation seems to offer a promising approach. Tocilizumab, a monoclonal antibody against IL-6, recently emerged as an alternative treatment for COVID-19 patients with a documented or cytokine storm [36]. Reports and single-center experiences [37, 38] have been documented and its actual efficacy is going to be assessed by dedicated investigations [NCT04317092].

## Laboratory disorders in SARS-CoV-2 infection vs V-V ECMO

Table 1 lists the major laboratory changes observed both during ECMO therapy and COVID-19 infection. Of importance is the multiplication of alterations induced by these V-V ECMO circuits caused by SARS-CoV-2 infection. Of particular importance are the fluctuations in hematological, biochemical, and coagulation level characteristics.

**Table 1:** Comparison of hematological and biochemical parameters in V-V ECMO and SARS-CoV-2 induced ARDS

	V-V ECMO	SARS-CoV-2 ARDS
**Hematological findings**		
White blood cell count	Initial ↑	↑
Lymphocyte	↓	↓↓
Neutrophil	Initial ↑	↑
Neutrophil activation	Initial ↑	?
Monocyte	Initial ↑	∽
CD3^+^, CD4^+^, CD8^+^, T cells	↓	↓↓
Natural killer cells	↓	∽ ↓
Neutrophil to lymphocyte ratio	↑	↑
Hemoglobin and red blood cell count	↓	∽
Platelet count	↓	↓∽
**Coagulation and anticoagulation**		
Platelet activation	↑	?
Platelet aggregation	↓	?
Platelet activation factor	↑	?
Heparin-induced thrombocytopenia	↑	?
Von Willebrand factor	↓	?
D-dimer	↑	↑↑
Fibrin degradation products		↑↑
Activated partial thromboplastin time	↑	∽
Prothrombin time		∽
Thrombospondin	↓	?
Fibronectin	↓	?
Thrombin	↑	?
Fibrinogen	Initial ↓	↑
High molecular weight kininogen	↑	?
Prekallikrein	↓	?
Kallikrein	↑	?
FVIII	↓	?
FX	↑	?
FXI	↓	?
FXIa	↑	?
FXII	↓	?
FXIIa	Rapid ↑	?
FXIII	↓	?
Antithrombin	Initially ↓ (UFH)	↓
C-protein	↑↓	?
Activated clotting time	↑	?
R-time thromboelastography	↑	?
**Inflammatory response**		
Tissue factor	↑∽	?
Bradykinin	↑	?
TNF-alpha	↑	∽↑
IFN-gamma	?	↑ (4–6 days after presentation)
IL-1-beta	↑	↓↓
IL-2	?	↑ 4–6 days after presentation
IL-2R	?	↑
IL-4	?	∽
IL-6	↑	↑↑
IL-8	↑	?
IL-10	↑	↑
IgE	↓	?
IgA	?	∽
IgG	?	∽↑
IgM	?	∽
Complement	∽↑	∽

*UFH* unfractionated heparin

The references to support the above table are listed as supplementary S1-S82

Analysis of the available literature shows that thrombocytopenia in patients infected with SARS-CoV-2 is a relatively rare phenomenon [16, 39–41]. Nevertheless, the biggest study to date, which included 1099 COVID-19 patients, showed that those treated in intensive care units had a reduced platelet count [3]. In a study underlining differences of coagulation features between severe pneumonia induced by SARS-CoV-2 and non-SARS-CoV-2, the platelet count of the COVID group was significantly higher than that of non-COVID patients [42]. A role of the increased thrombopoietin levels following pulmonary inflammation has been proposed, exacerbating more severe inflammation reaction and hypercoagulability. Platelets are produced by megakaryocytes in the bone marrow, and a variety of cytokines, including IL3, IL-6, IL-9, and IL-11, are able to trigger their production [43]. Moreover, clinical and experimental evidence indicates that platelets are a source of microvesicles with a strong pro-inflammatory potential. On the other hand, some authors have shown that platelets increased first and then decreased in severe COVID patients during the hospital stay and therefore speculated that the changes in platelets in the treatment course may correlate with the progression and prognosis of COVID-19 [44]. The relationship between the low number of platelets and the severity of the disease was also reported in a meta-analysis of 9 studies of 1779 patients [45]. Again, patients with severe disease had a lower platelet count [46]. In addition, determination of platelet counts is recommended for all patients in light of the published International Society on Thrombosis and Haemostasis (ISTH) guidelines [47]. It should be stressed, however, that platelet monitoring should be combined in clinical practice with an assessment of their functioning. Unfortunately, the current data do not allow conclusions on this issue. Thrombocytopenia, on the other hand, is one of the many complications of ECMO [48–50]. It is estimated that approximately 1 in 5 patients may experience severe thrombocytopenia (platelets < 50 × 109/L) [48]. This complication appears to increase with the duration of ECMO; however, the mechanism of thrombocytopenia is not as simple as it initially appears. Abrams et al. [50] showed that low platelet count is not related to the duration of ECMO, but rather to platelet count and disease severity at the time of cannulation. In the pathogenesis of thrombocytopenia during ECMO, the possibility of heparin-induced thrombocytopenia (HIT) should also be considered [51]. Interestingly, patients with HIT on VA-ECMO are characterized by much more severe thrombocytopenia compared to VV-ECMO [51]. In addition, the severity of this complication may depend on the system used in ECMO—Priming Reduced Extracorporeal Circulation Setup (PRECiSe) patients have lower platelet count than minimal extracorporeal circuit (MECC) cases [52]. Functional analysis of platelets also shows that platelet activation depends on a factor stimulating this process [52].

It is widely acknowledged that the majority of COVID-19 patients, especially those with severe disease, are characterized by lymphocytopenia. This laboratory symptom is observed mostly in adult patients, much less often to children [45], and may predict COVID-19 severity [53]. The second important remark is that the reduced number of lymphocytes is also a common feature of diseases caused by other coronaviruses, including SARS [54] and MERS [55]. Notably, the currently available data strongly indicates that lymphocytopenia is dynamically modulated by the intensification of local and systemic inflammation, direct infection of lymphocytes, and destruction of lymphoid organs [55, 56]. In addition, treatment with glucocorticosteroids may cause lymphocytopenia in some cases [57].

Coagulopathies of diverse etiologies were described in COVID-19 patients; of importance is the augmented risk of venous thromboembolism (VTE). Though there are no published case series thus far, there exist reports of abnormal coagulation parameters in hospitalized patients with severe COVID-19 disease. In a multicenter retrospective cohort study from China, elevated D-dimer levels (> 1 g/L) were strongly associated with in-hospital death, even after multivariable adjustment (OR 18.4, 95% CI 2.6–128.6; *p* = 0.003) [32]. In the cohort study by Wu et al. [58] involving 201 patients with confirmed COVID-19 pneumonia, risk factors associated with the development of ARDS and progression from ARDS to death included among others coagulation dysfunction. For patients with ARDS who died, coagulation function indices (D-dimer [difference, 2.10 μg/mL; 95% CI, 0.89–5.27 μg/mL; *p* = 0.001]) were significantly elevated compared with patients with ARDS who survived; elevated D-dimers were elevated and prognostic of worse outcome in other reports as well. In another study comparing COVID-19 survivors to non-survivors, non-survivors had significantly higher D-dimer and fibrin degradation product (FDP) levels and 71.4% of non-survivors met clinical criteria for disseminated intravascular coagulation (DIC) during the course of their disease [17].

Other laboratory parameters for the routine assessment of blood coagulation appear to be normal in COVID-19 patients regardless of the severity of infection, except for a single case of prolonged prothrombin time (PT) [39] and reduced activated partial thromboplastin time (aPTT) [59] in severe SARS-CoV-2–infected cases.

While the optimal anticoagulation regimens to prevent VTE and DIC in the setting of critically ill, immobilized COVID-19 patients remain unknown, V-V ECMO is a double-edged sword; on one side, it promotes thrombosis and hypercoagulable state; on the other, ECMO circuits are eliminating coagulation factors binding them irreversibly to surface coating material. In fact, iv. administered unfractionated heparin (UFH) which is required for the ECMO run [60] is further aggravating anticoagulatory state by interplaying with antithrombin on many levels. Anti-inflammatory functions of AT are partly mediated through inhibition of thrombin that reduces platelet activation, neutrophil–endothelial cell interactions, and endothelial upregulations [25]. In patients on ECMO, acquired AT deficiency is a result of hemodilution, blood coagulation activation, and consumption due to the use of UFH [61]. The binding of AT to UFH competes with the binding of AT to endothelial GAGs, thus increasing the likelihood of inflammation-related complications. In this light, low levels of AT can increase the risk of either thrombotic or hemorrhagic complications, the first because of the reduced effect of heparin, and the second due to relevant concomitant inflammatory response, organ damage, and concomitant coagulation factor consumption [30, 62–64]. While several authors have reported a relevant reduction in mortality with higher dosage of heparin in COVID-19, it is too early to gather definitive conclusions but a deep attention to the coagulation profile during ECMO should be considered. As a desired positive “side effect” of ECMO support, the use of high-dosage anticoagulation as well as an attitude to a thorough coagulation profile study may contribute to a better outcome in COVID-19.

## Evidence summary of available reports on COVID-19 and ECMO

Table 2 summarizes the outcomes gathered from experiences with ECMO in COVID-19; a database search for valid records was conducted according to PRISMA guidelines until April 5; keywords used were ECLS, ECMO, COVID-19, and SARS-CoV-2; studies mentioning ECMO treatment in COVID-19 were included; case reports were not considered. Eleven studies were included [3, 16, 17, 31, 32, 39, 58, 65–68]. Potential overlap of patient populations cannot be excluded. Of 2884 COVID-19 patients, 440 (15.5%) developed ARDS and 401 (14.1%) were transferred to ICU; forty-two patients (1.5%) were treated with ECMO; in an overall cohort, observed mortality was 273 (9.6%). Outcome data of ECMO patients are incomplete with regard to ECMO configurations, duration, and indication. Shen et al. [67] demonstrated favorable outcome in case series of 5 critically ill patients, one of which had ECMO implanted and was successfully weaned 5 days after transfusion of convalescent plasma with a SARS-CoV-2–specific antibody. On the other hand, in the study by Yang et al. [68] who compared clinical characteristics and outcomes in patients with severe COVID-19, five (83%) of six patients receiving ECMO died. Ruan et al. [31] and Zhou et al. [32] reported 100% mortality for ECMO patients. Although these samples were small, and specific baseline characteristics and disease courses were almost unknown, the studies raise concerns about potential harms of ECMO therapy for COVID-19. This is, indeed, further reflected in guideline recommendations; “Clinical management of severe acute respiratory infection when Novel coronavirus (nCoV) infection is suspected” Interim Guidance document by WHO recommends to “consider referral patients with refractory hypoxemia despite lung-protective ventilation in settings with access to expertise in ECLS” [69]. Similarly, the United States Center for Disease Control provides interim guidance for clinical management of COVID-19 patients with and without ARDS: “Where ECLS expertise is available, ECLS should be considered according to the standard management algorithm for ARDS in supporting patients with viral lower respiratory tract infection” [70]. However, clearly, at this time, there is little worldwide experience with using ECLS to support COVID-19 patients; ELSO leaders have discussed the potential role of ECMO for COVID-19 patients in a recent *JAMA* Viewpoint [71] stating that ECMO is not a therapy to be rushed to the frontline when all resources are stretched in a pandemic and pointing to problems with proper ECMO referral and management in centers less well experienced in these therapies. Support with ECMO is further not available in many low- and middle-income countries; therefore, ECMO might not seem to gain as much of a priority as personal protective equipment, correct management, diagnosis and quarantine, oxygen therapy alone, and mechanical ventilation in first stance [72].

**Table 2:** The outcomes gathered from experiences with ECMO in COVID-19

Study	Type	Location	*N*	ICU admission	ARDS	ECMO	Overall mortality	ECMO mortality
Chen et al. [65]	Retrospective observational	Wuhan Jinyintan Hospital	99	23 (23.2%)	17 (17.2%)	3 (3.0%)	11 (11.1%)	NA
Guan et al. [3]	Cross-sectional	552 hospitals in 30 provinces, autonomous regions, and municipalities in mainland China	1099	55 (5.0%)	37 (3.4%)	5 (0.5%)	15 (1.4%)	NA
Huang et al. [39]	Cross-sectional	Jin Yin-tan Hospital, Wuhan, China	41	13 (31.7%)	12 (29.3%)	2 (4.9%)	6 (14.6%)	NA
Liu et al. [66]	Retrospective observational	Nine tertiary hospitals in Hubei	137	NA	34 (24.8%)*	0 (0.0%)	16 (11.7%)	NA
Ruan et al. [31]	Retrospective multicenter study	Jin Yin-tan Hospital and Tongji Hospital	150	41 (27.3%)	62 (41.3%)	7 (4.7%)	68 (45.3%)	7 (100%)
Shen et al. [67]	Case series	Shenzhen Third People’s Hospital in Shenzhen, China	5	5 (100%)	5 (100%)	1 (20.0%)	0 (0.0%)	0 (0.0%)
Tang et al. [17]	Retrospective case-control study	Wuhan Pulmonary Hospital	73	73 (100%)	73 (100%)	10 (13.7%)	21 (28.3%)	NA
Wang et al. [16]	Case series	Zhongnan Hospital of Wuhan University in Wuhan, China	138	36 (26.1%)	22 (15.9%)	4 (2.9%)	6 (4.3%)	NA
Wu et al. [58]	Retrospective cohort study	Wuhan Jinyintan Hospital	201	53 (26.4%)	84 (41.8%)	1 (0.5%)	44 (21.9%)	NA
Yang et al. [68]	Retrospective observational	Wuhan Jin Yin-tan hospital (Wuhan, China)	710	52 (7.3%)	35 (4.9%)	6 (0.8%)	32 (4.5%)	5 (83.3%)
Zhou et al. [32]	Retrospective cohort study	Jinyintan Hospital and Wuhan Pulmonary Hospital	191	50 (26.2%)	59 (30.9%)	3 (1.6%)	54 (28.3%)	3 (100.0%)

*Non-invasive ventilation

Finally, ELSO will continue to collect data through member centers through the ELSO Registry and provide recommendations as additional information becomes available from ongoing studies [73].

## Conclusions

Because ECMO therapy and COVID-19 itself are associated with certain, often synergistic changes in hematological and inflammatory status of the patients, the efficacy of ECMO is largely dependent on centers’ experience with such therapies.

## Supplementary information

**Additional file 1.** Supplementary references.

## Data Availability

All data generated or analyzed during this study are included in this published article [and its supplementary information files].
