# Supplementary material for: COVID-19 and ECMO: the interplay between coagulation and inflammation—a narrative review
Source: Crit Care. 2020 May 8;24:205. doi: 10.1186/s13054-020-02925-3 (PMC7209766; doi:10.1186/s13054-020-02925-3)
Supplement: Supplementary file 1 — Additional file 1. Supplementary references. [file 13054_2020_2925_MOESM1_ESM.docx]

**COVID-19 and ECMO. The interplay between coagulation and inflammation.**

**A narrative review.**

Supplementary material

**Supplementary references:**

1. Millar JE, Fanning JP, McDonald CI, McAuley DF, Fraser JF. The inflammatory response to extracorporeal membrane oxygenation (ecmo): A review of the pathophysiology. Crit Care. 2016;20:387

2. Wendel HP, Scheule AM, Eckstein FS, Ziemer G. Haemocompatibility of paediatric membrane oxygenators with heparin-coated surfaces. Perfusion. 1999;14:21-28

3. Wachtfogel YT, Hack CE, Nuijens JH, Kettner C, Reilly TM, Knabb RM, Bischoff R, Tschesche H, Wenzel H, Kucich U, et al. Selective kallikrein inhibitors alter human neutrophil elastase release during extracorporeal circulation. Am J Physiol. 1995;268:H1352-1357

4. Rodell TC, Naidoo Y, Bhoola KD. Role of kinins in inflammatory responses. Clin Immunotherapeutics. 2012;3(5):352–61.

5. Cugno M, Nussberger J, Biglioli P, Giovagnoni MG, Gardinali M, Agostoni A. Cardiopulmonary bypass increases plasma bradykinin concentrations. Immunopharmacology. 1999;43(2–3):145–7.

6. Morgan EN, Pohlman TH, Vocelka C, Farr A, Lindley G, Chandler W, Griscavage-Ennis JM, Verrier ED. Nuclear factor kappab mediates a procoagulant response in monocytes during extracorporeal circulation. J Thorac Cardiovasc Surg. 2003;125:165-171

7. Kappelmayer J, Bernabei A, Edmunds LH, Edgington TS, Colman RW. Tissue factor is expressed on monocytes during simulated extracorporeal circulation. Circ Res. 1993;72(5):1075–81.

8. Barstad RM, ØVrum E, Ringdal M-AL, ØYstese R, Hamers MJAG, Veiby OP, Rolfsen T, Stephens RW, Sakariassen KS. Induction of monocyte tissue factor procoagulant activity during coronary artery bypass surgery is reduced with heparin-coated extracorporeal circuit. Br J Haematol. 1996;94(3):517–25.

9. Szotowski B, Antoniak S, Poller W, Schultheiss H-P, Rauch U. Procoagulant soluble tissue factor is released from endothelial cells in response to inflammatory cytokines. Circ Res. 2005;96(12):1233–9.

10. Prescott SM, Zimmerman GA, McIntyre TM. Human endothelial cells in culture produce platelet-activating factor (1-alkyl-2-acetyl-sn-glycero-3- phosphocholine) when stimulated with thrombin. Proc Natl Acad Sci USA. 1984;81(11):3534–8.

11. Johnson K, Choi Y, DeGroot E, Samuels I, Creasey A, Aarden L. Potential mechanisms for a proinflammatory vascular cytokine response to coagulation activation. J Immunol. 1998;160:5130-5135

12. Kaplanski G, Fabrigoule M, Boulay V, Dinarello CA, Bongrand P, Kaplanski S, Farnarier C. Thrombin induces endothelial type ii activation in vitro: Il-1 and tnf-alpha-independent il-8 secretion and e-selectin expression. J Immunol. 1997;158:5435-5441

13. Dery O, Corvera CU, Steinhoff M, Bunnett NW. Proteinase-activated receptors: Novel mechanisms of signaling by serine proteases. Am J Physiol. 1998;274:C1429-1452

14. Vu TK, Hung DT, Wheaton VI, Coughlin SR. Molecular cloning of a functional thrombin receptor reveals a novel proteolytic mechanism of receptor activation. Cell. 1991;64:1057-1068

15. Sugama Y, Tiruppathi C, offakidevi K, Andersen TT, Fenton JW, 2nd, Malik AB. Thrombin-induced expression of endothelial p-selectin and intercellular adhesion molecule-1: A mechanism for stabilizing neutrophil adhesion. J Cell Biol. 1992;119:935-944

16. Levy JH, Tanaka KA. Inflammatory response to cardiopulmonary bypass. Ann Thorac Surg. 2003;75:S715-720

17. Gemmell CH, Ramirez SM, Yeo EL, Sefton MV. Platelet activation in whole blood by artificial surfaces: Identification of platelet-derived microparticles and activated platelet binding to leukocytes as material-induced activation events. J Lab Clin Med. 1995;125:276-287

18. Cheung PY, Sawicki G, Salas E, Etches PC, Schulz R, Radomski MW. The mechanisms of platelet dysfunction during extracorporeal membrane oxygenation in critically ill neonates. Crit Care Med. 2000;28:2584-2590

19. Fernandez-Patron C, Martinez-Cuesta MA, Salas E, Sawicki G, Wozniak M, Radomski MW, Davidge ST. Differential regulation of platelet aggregation by matrix metalloproteinases-9 and -2. Thromb Haemost. 1999;82:1730-1735

20. Maugeri N, Brambilla M, Camera M, Carbone A, Tremoli E, Donati MB, De Gaetano G, Cerletti C. Human polymorphonuclear leukocytes produce and express functional tissue factor upon stimulation1. J Thromb Haemost. 2006; 4(6):1323–30.

21. Plotz FB, van Oeveren W, Bartlett RH, Wildevuur CR. Blood activation during neonatal extracorporeal life support. J Thorac Cardiovasc Surg. 1993;105:823-832

22. Dunkelberger JR, Song W-C. Complement and its role in innate and adaptive immune responses. Cell Res. 2009;20(1):34–50.

23. Lindholm L, Westerberg M, Bengtsson A, Ekroth R, Jensen E, Jeppsson A. A closed perfusion system with heparin coating and centrifugal pump improves cardiopulmonary bypass biocompatibility in elderly patients. Ann Thorac Surg. 2004;78(6):2131–8. discussion 2138.

24. Morgan IS, Codispoti M, Sanger K, Mankad PS. Superiority of centrifugal pump over roller pump in paediatric cardiac surgery: prospective randomised trial. Eur J Cardiothorac Surg. 1998;13(5):526–32.

25. Hein E, Munthe-Fog L, Thiara AS, Fiane AE, Mollnes TE, Garred P. Heparin- coated cardiopulmonary bypass circuits selectively deplete the pattern recognition molecule ficolin-2 of the lectin complement pathway in vivo. Clin Exp Immunol. 2015;179(2):294–9.

26. Vallhonrat H, Swinford RD, Ingelfinger JR, Williams WW, Ryan DP, Tolkoff-Rubin N, Cosimi AB, Pascual M. Rapid activation of the alternative pathway of complement by extracorporeal membrane oxygenation. ASAIO J. 1999;45:113-114

27. Warren OJ, Smith AJ, Alexiou C, Rogers PL, Jawad N, Vincent C, Darzi AW, Athanasiou T. The inflammatory response to cardiopulmonary bypass: Part 1--mechanisms of pathogenesis. J Cardiothorac Vasc Anesth. 2009;23:223-231

28. Perkins GD, Nathani N, McAuley DF, Gao F, Thickett DR. In vitro and in vivo effects of salbutamol on neutrophil function in acute lung injury. Thorax. 2007;62:36-42

29. Kotani N, Hashimoto H, Sessler DI, Muraoka M, Wang JS, O'Connor MF, Matsuki A. Neutrophil number and interleukin-8 and elastase concentrations in bronchoalveolar lavage fluid correlate with decreased arterial oxygenation after cardiopulmonary bypass. Anesth Analg. 2000;90:1046-1051

30. Kiaii B, Fox S, Swinamer SA, Rayman R, Higgins J, Cleland A, Fernandes P, MacDonald J, Dobkowski WB, Stitt LW, Novick RJ, Singh B, Bureau Y, Summers K. The early inflammatory response in a mini-cardiopulmonary bypass system: A prospective randomized study. Innovations (Phila). 2012;7:23-32

31. Hocker JR, Wellhausen SR, Ward RA, Simpson PM, Cook LN. Effect of extracorporeal membrane oxygenation on leukocyte function in neonates. Artif Organs. 1991;15(1):23–8.

32. DePuydt LE, Schuit KE, Smith SD. Effect of extracorporeal membrane oxygenation on neutrophil function in neonates. Crit Care Med. 1993; 21(9):1324–7.

33. Graulich J, Walzog B, Marcinkowski M, Bauer K, Kossel H, Fuhrmann G, Buhrer C, Gaehtgens P, Versmold HT. Leukocyte and endothelial activation in a laboratory model of extracorporeal membrane oxygenation (ECMO). Pediatr Res. 2000;48(5):679–84.

34. McIlwain RB, Timpa JG, Kurundkar AR, Holt DW, Kelly DR, Hartman YE, Neel ML, Karnatak RK, Schelonka RL, Anantharamaiah GM, Killingsworth CR, Maheshwari A. Plasma concentrations of inflammatory cytokines rise rapidly during ecmo-related sirs due to the release of preformed stores in the intestine. Lab Invest. 2010;90:128-139

35. Fortenberry JD, Bhardwaj V, Niemer P, Cornish JD, Wright JA, Bland L. Neutrophil and cytokine activation with neonatal extracorporeal membrane oxygenation. J Pediatr. 1996;128:670-678

36. Adrian K, Mellgren K, Skogby M, Friberg LG, Mellgren G, Wadenvik H. Cytokine release during long-term extracorporeal circulation in an experimental model. Artif Organs. 1998;22(10):859–63.

37. Yimin H, Wenkui Y, Jialiang S, Qiyi C, Juanhong S, Zhiliang L, Changsheng H, Ning L, Jieshou L. Effects of continuous renal replacement therapy on renal inflammatory cytokines during extracorporeal membrane oxygenation in a porcine model. J Cardiothorac Surg. 2013;8:113

38. Shi J, Chen Q, Yu W, Shen J, Gong J, He C, Hu Y, Zhang J, Gao T, Xi F, Li J. Continuous renal replacement therapy reduces the systemic and pulmonary inflammation induced by venovenous extracorporeal membrane oxygenation in a porcine model. Artif Organs. 2014;38:215-223

39. Jialiang S, Juanhong S, Qiyi C, Wenkui Y, Changsheng H, Yimin H, Juanjuan Z, Tao G, Fengchan X, Jieshou L. In-line hemofiltration minimized extracorporeal membrane oxygenation-related inflammation in a porcine model. Perfusion. 2014;29(6):526–33.

40. Hong TH, Kuo SW, Hu FC, Ko WJ, Hsu LM, Huang SC, Yang YW, Yu SL, Chen YS. Do interleukin-10 and superoxide ions predict outcomes of cardiac extracorporeal membrane oxygenation patients? Antioxid Redox Signal. 2014;20(1):60–8.

41. Besser MW, Klein AA. The coagulopathy of cardiopulmonary bypass. Crit Rev Clin Lab Sci. 2010;47:197-212.

42. Da Q, Teruya M, Guchhait P, Teruya J, Olson JS, Cruz MA. Free hemoglobin increases von Willebrand factor-mediated platelet adhesion in vitro: implications for circulatory devices. Blood. 2015;126:2338-41.

43. Panigada M, Artoni A, Passamonti SM, Maino A, Mietto C, L'Acqua C, Cressoni M, Boscolo M, Tripodi A, Bucciarelli P, Gattinoni L, Martinelli I. Hemostasis changes during veno-venous extracorporeal membrane oxygenation for respiratory support in adults. Minerva Anestesiol. 2016;82:170-179

44. Abrams D, Baldwin MR, Champion M, Agerstrand C, Eisenberger A, Bacchetta M, Brodie D. Thrombocytopenia and extracorporeal membrane oxygenation in adults with acute respiratory failure: A cohort study. Intensive Care Med. 2016;42:844-852

45. Kalbhenn J, Wittau N, Schmutz A, Zieger B, Schmidt R. Identification of acquired coagulation disorders and effects of target-controlled coagulation factor substitution on the incidence and severity of spontaneous intracranial bleeding during veno-venous ecmo therapy. Perfusion. 2015;30:675-682

46. Kalbhenn J, Schmidt R, Nakamura L, Schelling J, Rosenfelder S, Zieger B. Early diagnosis of acquired von willebrand syndrome (avws) is elementary for clinical practice in patients treated with ecmo therapy. J Atheroscler Thromb. 2015;22:265-271

47. Heilmann C, Geisen U, Beyersdorf F, Nakamura L, Benk C, Trummer G, Berchtold-Herz M, Schlensak C, Zieger B. Acquired von willebrand syndrome in patients with extracorporeal life support (ecls). Intensive Care Med. 2012;38:62-68

48. Pieri M, Turla OG, Calabro MG, Ruggeri L, Agracheva N, Zangrillo A, Pappalardo F. A new phosphorylcholine-coated polymethylpentene oxygenator for extracorporeal membrane oxygenation: A preliminary experience. Perfusion. 2013;28:132-137

49. Baghai M, Tamura N, Beyersdorf F, Henze M, Prucker O, Ruhe J, Goto S, Zieger B, Heilmann C. Platelet repellent properties of hydrogel coatings on polyurethane-coated glass surfaces. ASAIO J. 2014;60:587-593

50. Linneweber J, Dohmen PM, Kertzscher U, Affeld K, Nose Y, Konertz W. The effect of surface roughness on activation of the coagulation system and platelet adhesion in rotary blood pumps. Artif Organs. 2007;31:345-351

51. Annich GM, Lynch WR, MacLaren G, Wilson JM, Bartlet RH. ECMO: Extracorporeal Cardiopulmonary Support in Critical Care (The “Red Book”). ELSO, 2013.

52. Fina D, Matteucci M, Jiritano F, Meani P, Lo Coco V, Kowalewski M, Maessen J, Guazzi M, Ballotta A, Ranucci M, Lorusso R. Extracorporeal membrane oxygenation without therapeutic anticoagulation in adults: A systematic review of the current literature. Int J Artif Organs. 2020:391398820904372

53. Malfertheiner MV, Philipp A, Lubnow M, Zeman F, Enger TB, Bein T, Lunz D, Schmid C, Muller T, Lehle K. Hemostatic changes during extracorporeal membrane oxygenation: A prospective randomized clinical trial comparing three different extracorporeal membrane oxygenation systems. Crit Care Med. 2016;44:747-754

54. Koning NJ, Vonk AB, Vink H, Boer C. Side-by-Side Alterations in Glycocalyx Thickness and Perfused Microvascular Density During Acute Microcirculatory Alterations in Cardiac Surgery. Microcirculation. 2016;23:69-74.

55. Ranucci M, Baryshnikova E, Isgro G, Carlucci C, Cotza M, Carboni G, Ballotta A. Heparin-like effect in postcardiotomy extracorporeal membrane oxygenation patients. Crit Care. 2014;18:504

56. Glick D, Dzierba AL, Abrams D, Muir J, Eisenberger A, Diuguid D, Abel E, Agerstrand C, Bacchetta M, Brodie D. Clinically suspected heparin-induced thrombocytopenia during extracorporeal membrane oxygenation. J Crit Care. 2015;30:1190-1194

57. Glick D, Dzierba AL, Abrams D, Muir J, Eisenberger A, Diuguid D, Abel E, Agerstrand C, Bacchetta M, Brodie D. Clinically suspected heparin-induced thrombocytopenia during extracorporeal membrane oxygenation. J Crit Care. 2015;30:1190-1194

58. Wilm J, Philipp A, Muller T, Bredthauer A, Gleich O, Schmid C, Lehle K. Leukocyte adhesion as an indicator of oxygenator thrombosis during extracorporeal membrane oxygenation therapy? ASAIO J. 2018;64:24-30

59. Doyle AJ, Hunt BJ. Current understanding of how extracorporeal membrane oxygenators activate haemostasis and other blood components. Front Med (Lausanne). 2018;5:352

60. Yost G, Bhat G, Pappas P, Tatooles A. The neutrophil to lymphocyte ratio in patients supported with extracorporeal membrane oxygenation. Perfusion. 2018;33:562-567

61. Francischetti IMB, Szymanski J, Rodriguez D, Heo M, Wolgast LR. Laboratory and clinical predictors of 30-day survival for patients on extracorporeal membrane oxygenation (ecmo): 8-year experience at albert einstein college of medicine, montefiore medical center. J Crit Care. 2017;40:136-144

62. Qian Y, Xie H, Tian R, Lu J, Jin W, Wang R. [clinical significance of early immunological paralysis in patients with severe h1n1 influenza a]. Zhonghua Wei Zhong Bing Ji Jiu Yi Xue. 2017;29:581-585

63. Oliver WC. Anticoagulation and coagulation management for ecmo. Semin Cardiothorac Vasc Anesth. 2009;13:154-175

64. Esper SA, Levy JH, Waters JH, Welsby IJ. Extracorporeal membrane oxygenation in the adult: A review of anticoagulation monitoring and transfusion. Anesth Analg. 2014;118:731-743

65. Bembea MM, Schwartz JM, Shah N, Colantuoni E, Lehmann CU, Kickler T, Pronovost P, Strouse JJ. Anticoagulation monitoring during pediatric extracorporeal membrane oxygenation. ASAIO J. 2013;59:63-68

66. Arnold P, Jackson S, Wallis J, Smith J, Bolton D, Haynes S. Coagulation factor activity during neonatal extra-corporeal membrane oxygenation. Intensive Care Med. 2001;27:1395-1400

67. Levi M, Meijers JC. Dic: Which laboratory tests are most useful. Blood Rev. 2011;25:33-37

68. Bernard GR, Vincent JL, Laterre PF, LaRosa SP, Dhainaut JF, Lopez-Rodriguez A, Steingrub JS, Garber GE, Helterbrand JD, Ely EW, Fisher CJ, Jr., Recombinant human protein CWEiSSsg. Efficacy and safety of recombinant human activated protein c for severe sepsis. N Engl J Med. 2001;344:699-709

69. Lou S, MacLaren G, Best D, Delzoppo C, Butt W. Hemolysis in pediatric patients receiving centrifugal-pump extracorporeal membrane oxygenation: Prevalence, risk factors, and outcomes. Crit Care Med. 2014;42:1213-1220

70. Okochi S, Cheung EW, Barton S, Zenilman A, Shakoor A, Street C, Streltsova S, Chan C, Brewer MP, Middlesworth W. An analysis of risk factors for hemolysis in children on extracorporeal membrane oxygenation. Pediatr Crit Care Med. 2018;19:1059-1066

71. Prakash S, Wiersema UF, Bihari S, Roxby D. Discordance between ROTEM(R) clotting time and conventional tests during unfractionated heparin-based anticoagulation in intensive care patients on extracorporeal membrane oxygenation. Anaesth Intensive Care. 2016;44(1):85–92.

72 Huang C, Wang Y, Li X, Ren L, Zhao J, Hu Y, Zhang L, Fan G, Xu J, Gu X, Cheng Z, Yu T, Xia J, Wei Y, Wu W, Xie X, Yin W, Li H, Liu M, Xiao Y, Gao H, Guo L, Xie J, Wang G, Jiang R, Gao Z, Jin Q, Wang J, Cao B. Clinical features of patients infected with 2019 novel coronavirus in Wuhan, China. Lancet. 2020;395(10223):497-506.

73. Wang D, Hu B, Hu C, Zhu F, Liu X, Zhang J, Wang B, Xiang H, Cheng Z, Xiong Y, Zhao Y, Li Y, Wang X, Peng Z. Clinical characteristics of 138 hospitalized patients with 2019 novel coronavirus-infected pneumonia in Wuhan, China. JAMA. 2020. doi: 10.1001/jama.2020.1585.

74. Guan WJ, Ni ZY, Hu Y, Liang WH, Ou CQ, He JX, Liu L, Shan H, Lei CL, Hui DSC, Du B, Li LJ, Zeng G, Yuen KY, Chen RC, Tang CL, Wang T, Chen PY, Xiang J, Li SY, Wang JL, Liang ZJ, Peng YX, Wei L, Liu Y, Hu YH, Peng P, Wang JM, Liu JY, Chen Z, Li G, Zheng ZJ, Qiu SQ, Luo J, Ye CJ, Zhu SY, Zhong NS; China Medical Treatment Expert Group for Covid-19. Clinical characteristics of coronavirus disease 2019 in China. N Engl J Med. 2020. doi: 10.1056/NEJMoa2002032.

75. Chang D, Lin M, Wei L, Xie L, Zhu G, Dela Cruz CS, Sharma L. Epidemiologic and clinical characteristics of novel coronavirus infections involving 13 patients outside Wuhan, China. JAMA. 2020;323(11):1092-1093.

76. Liu J, Li S, Liu J, Liang B, Wang X, Wang H, et al. Longitudinal characteristics of lymphocyte responses and cytokine profiles in the peripheral blood of SARS-CoV-2 infected patients. medRxiv. 2020. doi: 10.1101/2020.02.16.20023671.

77. Chen G, Wu D, Guo W, Cao Y, Huang D, Wang H, Wang T, Zhang X, Chen H, Yu H, Zhang X, Zhang M, Wu S, Song J, Chen T, Han M, Li S, Luo X, Zhao J, Ning Q. Clinical and immunologic features in severe and moderate Coronavirus Disease 2019. J Clin Invest. 2020. doi: 10.1172/JCI137244.

78. Liu J, Liu Y, Xiang P, Pu L, Xiong H, Li C, et al. Neutrophil-to-lymphocyte ratio predicts severe illness patients with 2019 novel coronavirus in the early stage. medRxiv. 2020. doi: 10.1101/2020.02.10.20021584.

79. Zhang B, Zhou X, Zhu C, Feng F, Qiu Y, Feng J, et al. Immune phenotyping based on neutrophil-to-lymphocyte ratio and IgG predicts disease severity and outcome for patients with COVID-19. medRxiv. 2020. doi: 10.1101/2020.03.12.20035048.

80. Tang N, Li D, Wang X, Sun Z. Abnormal coagulation parameters are associated with poor prognosis in patients with novel coronavirus pneumonia. J Thromb Haemost. 2020;18(4):844-847.

81. Han H, Yang L, Liu R, Liu F, Wu KL, Li J, Liu XH, Zhu CL. Prominent changes in blood coagulation of patients with SARS-CoV-2 infection. Clin Chem Lab Med. 2020. doi: 10.1515/cclm-2020-0188. [Epub ahead of print]

82. Tan L, Wang Q, Zhang D, Ding J, Huang Q, Tang YQ, et al. Lymphopenia predicts disease severity of COVID-19: a descriptive and predictive study. Signal Transduct Target Ther. 2020;5:33. doi: 10.1038/s41392-020-0148-4.
